# Neuroimmune crosstalk and its impact on cancer therapy and research

**DOI:** 10.1007/s12672-022-00547-5

**Published:** 2022-08-23

**Authors:** Iman Sharawy

**Affiliations:** Clinical Oncology and Nuclear Medicine, Faculty of Medicine, Ain Shams University, Cairo, Egypt

**Keywords:** Neuroimmune crosstalk, Tumor innervation, Exosomes, miRNA, Anti-neurogenic drugs, Drug repurposing, Oncolytic virus therapy

## Abstract

Cancer is a major health problem as it is the first or second leading cause of death worldwide. The global cancer burden is expected to rise 47% relative to 2020 cancer incidence. Recently, the fields of neuroscience, neuroimmunology and oncology have elaborated the neuroimmune crosstalk role in tumor initiation, invasion, progression, and metastases. The nervous system exerts a broad impact on the tumor microenvironment by interacting with a complex network of cells such as stromal, endothelial, malignant cells and immune cells. This communication modulates cancer proliferation, invasion, metastasis, induce resistance to apoptosis and promote immune evasion. This paper has two aims, the first aim is to explain neuroimmune crosstalk in cancer, tumor innervation origin and peripheral nervous system, exosomes, and miRNA roles. The second aim is to elaborate neuroimmune crosstalk impact on cancer therapy and research highlighting various potential novel strategies such as use of immune checkpoint inhibitors and anti-neurogenic drugs as single agents, drug repurposing, miRNA-based and si-RNA-based therapies, tumor denervation, cellular therapies, and oncolytic virus therapy.

## Cancer burden

Globally, an estimated 19.3 million new cancer cases and about 10.0 million cancer deaths occurred in 2020. The global cancer burden is expected to increase to 28.4 million cases in 2040, a 47% rise from 2020 [1]. According to the World Health Organization, cancer was the first or second leading cause of death under the age of 70 years in 112 of 183 countries and ranked third or fourth in other 23 countries in 2019 [2]. Recently, the fields of neuroscience, neuroimmunology and oncology have highlighted the role of neuroimmune crosstalk in tumor initiation, invasion, progression, and metastases resulting in novel therapeutic and research strategies. This paper has two aims, the first aim is to explain neuroimmune crosstalk in cancer, tumor innervation origin and the peripheral nervous system, exosomes, and micro-RNA (miRNA) roles. The second aim is to elaborate neuroimmune crosstalk impact on cancer therapy and research highlighting various potential novel strategies such as use of immune checkpoint inhibitors and anti-neurogenic drugs as single agents, drug repurposing, miRNA-based and small interfering RNA (si-RNA)-based therapies, cellular therapies, and oncolytic virus therapy.

## Tumor microenvironment

Both the central nervous system (including hypothalamic–pituitary–adrenal axis-corticotrophin-releasing hormone pathway) and the peripheral nervous system (PNS) exercise control over innate and adaptive immune response. Catecholamines secreted by sympathetic nervous system (SNS), whether secreted from the adrenal system and released in circulatory system or secreted from nerve cells within tumor microenvironment (TME), regulate almost all human organs’ functions [3].

TME is composed of stromal cells, immune cells, vasculature, nerve cells, and signaling molecules within an extracellular matrix (ECM). Malignant cells communicate either directly with TME cells or indirectly through mediators to modulate it. Although TME composition is heterogeneous, yet it serves similar biological roles across multiple cancers (modulate angiogenesis pathways, regulate the immune system, promote metabolic changes, and form pre-metastatic niches). Malignant cells secrete various molecules, which are grouped according to their function into neurotransmitters, neuropeptides, neurotrophins, their respective receptors and other molecules. Neurotrophic factors cause autocrine stimulation of tumor growth and increase tumor-nerve density [4, 5].

The immune cells found in the TME are myeloid-derived suppressor cells (MDSC), T and B regulatory cells, regulatory dendritic cells, tumor-associated macrophages, type 2 neutrophils, mast cells and other cells constituting the inflammatory milieu that either enhances or suppresses tumor growth. Malignant cells express high levels of programmed death-ligand 1 (PD-L1) allowing them to escape immune surveillance when they are bound to programmed death-1 (PD-1) expressed by T cells. Catecholamines secreted by nerve cells stimulate the gene encoding the β2-adrenergic receptor (b-AR) leading to stimulation of angiogenesis, lymphangiogenesis, enhancement of tumor metastatic pathways, and suppression of T cells, thus help in maintaining an immunosuppressive microenvironment [6].

## Neuroimmune crosstalk

The nervous system exerts a broad impact on TME by interacting with a complex network of cells such as stromal, endothelial, malignant cells and immune cells. This communication modulates cancer proliferation, invasion, metastasis, induce resistance to apoptosis and promote immune evasion. During neuroimmune crosstalk, cellular communication modes of immune cells (which include junctions, voltage-gated ion channels, cell surface, transmembrane receptors, ligands, membrane vesicles, transporters, and downstream signal transduction events) are affected by neurotransmitters and neuropeptides present in TME in an autocrine or paracrine fashion. As a result, neurotransmitters and neuropeptides are important in modulation of innate and adaptive immune responses. Anatomical proximity and molecular mechanisms of communication, including receptors and signaling messengers, facilitate neuroimmune crosstalk. Moreover, neuroimmune crosstalk occurs between nerves and cancer stem cells (CSC) enabling nerves to stimulate the CSC compartment and tissue homeostasis, via vital proliferative and survival pathways such as the Wnt, Notch, and Hedgehog pathways [7].

**Fig. 1:**
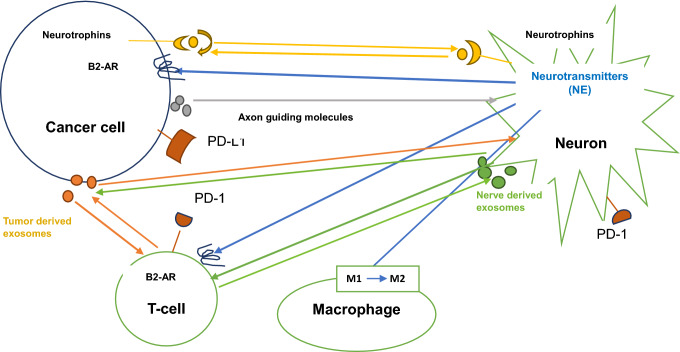
Neuroimmune crosstalk in TME. Neurotrophins, neurotransmitters as norepinephrine (NE), their specific receptors and axon guiding molecules modulate activity of cancer cells and other components of TME. NE released by nerve cells influences cancer cells, T cells and induces M2 activation of macrophages. Expression of PD-1 on T cells and nerve cells and expression of PD-L1 on cancer cells alter immunity within TME. Tumor derived exosomes and nerve derived exosomes act as mediators between nerves, immune cells, malignant cells and TME. Tumor-derived and nerve derived exosomes impact T cell function within TME, leading to an immune-suppressive environment

Not only nerve cells in TME impact malignant cells, but they impact nonmalignant cells to create a tumor-promoting environment. Nerve cells either directly stimulate malignant cells or indirectly through autocrine secretion of neurotransmitters (catecholamines and acetylcholine), neuropeptides, neurotrophic factors such as nerve growth factor (NGF), cytokines, chemokines, growth factors and checkpoint proteins as PD-1 (Fig. 1). Neurotransmitters and neuropeptides regulate cancer progression by their direct effects on malignant cells and/or modulation of immune cell function. Although the responsible mechanisms are tissue specific, neural signals within TME lead to cellular changes that enhance tumor growth, angiogenesis, immune modulation, and further recruitment of nerves to contribute to cancer progression. In addition, neural signaling modulates local TME to alter tumor immunity, angiogenesis, ECM, and stroma to promote cancer growth. Neural signaling may be altered by cancer cells through tumor-derived signals to develop metastatic niches that promote metastatic deposition and growth. In addition, nerve cells secrete neurotransmitters and neuropeptides that bind to specific receptors expressed on immune cells, thus modulating the immune system. Most immune cells such as tumor-infiltrating lymphocytes (TILs), dendritic cells, natural killer (NK) cells, macrophages, neutrophils, and MDSC express cell-surface neurotransmitter and neuropeptide receptors, activation/blockade of neurotransmission produces protumor and antitumor immune responses in a nerve-type and tumor type-dependent manner [8].

## Tumor innervation

Tumors actively recruit nerves into TME which stimulate tumorigenesis and tumor proliferation. Sources of neural contributions had been attributed to stimulation of preexisting regional nerves that innervate normal tissues to produce new tumor-directed axons (axonogenesis) via expression of neurotrophic factors such as NGF, the precursor of NGF, brain-derived neurotrophic factor, and others that are released by malignant cells [9]. These neurotrophins drive axonogenesis via stimulating tyrosine kinase receptors (expressed in nerve terminals) and binding to receptor tyrosine kinase (TRK) family [also called neurotrophic tyrosine receptor kinase (NTRK)]. NGF can also be secreted by tumor-associated immune cells and fibroblasts. As axonogenesis occur as an early event (present in pre-neoplastic lesions), it may contribute to initiation of cancer [10]. Alterations in neurotrophic factor signaling impact progression of precancerous lesions to invasive cancer by affecting tumor precursor cells and/or tissue innervation. Additionally, cancer cells secrete axon guidance molecules, which act in synergy with neurotrophic growth factors to enhance axonogenesis [11].

Another mechanism of tumor denervation is through neurogenesis i.e., increased number of neurons. Tumors can initiate their own innervation and attain a neural phenotype, through paracrine secretion, a phenomenon referred to as neurotrophic effect [12]. Lastly, tumor denervation can occur through recruitment of neural progenitor cells with high migration abilities, followed by differentiation into mature neural cells [10]. This mechanism may open a novel avenue for diagnosing, monitoring, and treating cancer through inhibiting neural progenitor cells’ migration to cancer cells and targeting neural progenitor cells in TME [13].

## Peripheral nervous system

Nerve fibers arising from PNS innervate most solid tumors (except brain and spinal cord tumors) and form a part of TME [14].

## Parasympathetic nervous system

Vagal immunomodulatory effects, also known as the cholinergic anti-inflammatory pathway, are mediated primarily by acetylcholine, which is the predominant neurotransmitter of the parasympathetic system that activates muscles, glands, and other organs, modulates primary tumor growth, and is implicated in tumor immunity process. The parasympathetic nervous system-derived acetylcholine, and other factors bind to specific receptors on malignant cells and promote cancer progression [15].

## Sympathetic nervous system

The SNS modulates b-AR signaling of immune cells in TME through local norepinephrine secretion or through circulating epinephrine/norepinephrine. b-adrenergic secretion can directly affect carcinogenesis signaling, inhibit DNA damage repair, downregulate p53-associated apoptosis, modulate various growth and survival pathways, and activate mesenchymal cell types present in TME such as fibroblasts, pericytes, mesenchymal stem cells, alter endothelial cells metabolism and activate an angiogenic switch either directly through endothelial cell activation, or indirectly by stimulating secretion of vascular endothelial growth factor (VEGF) [16].

## Schwann cells

Schwann cells are the major glial cell type in peripheral nervous system. They play a major role in peripheral nerves’ development, maintenance, function, and regeneration. During expansion, solid tumors injure and destroy nerves activating neurodegenerative and nerve repair functions of glial cells. Malignant cells affect Schwann cell function by reprogramming them to a repair-like phenotype (tumor-activated Schwann cells) which are potent promoters of TME. Tumor-induced Schwann cells reprogramming promotes cancer growth through extracellular matrix and local immune microenvironment alterations, the latter mechanism is an important factor in cancer development and progression. Schwann cells are involved in immune regulation, nerve maintenance and repair, neuropathic pain, repair of nonneuronal tissue, and cancer progression via direct effect on malignant cells, alterations in TME, and establishment of premalignant niche in various tissues [17].

## Role of exosomes

All cells secrete exosomes which are small, membrane-bound vesicles (30–150 nm) that act as a molecular signaling mechanism by which nerves induce tumorigenesis and control TME. Exosome content is dependent on cell of origin. As exosomes can travel short and long distances to reach their target cells, their cargo can be taken up to exert biological functions both locally (within the TME) and distantly (promotion of distant metastases) [18]. Consequently, exosomes act as mediators of oncogenic processes by potentiating cell–cell communication locally, and oncogenic processes distantly via establishing distant metastatic niches. Heterotypic cell–cell communication between malignant cells and nerves occurs through exosomes which induces tumor innervation and mediate neural regulation of TME. Additionally, this crosstalk may result in secretion of exosomes from malignant cells which package axonal guidance molecules inducing axonogenesis. Exosome-induced neural reprogramming occurs during cancer development, suggesting that cancer-derived extracellular vesicles have a role in axonogenesis [19]. Exosomes hold both diagnostic and therapeutic potential for cancer management through exosome-based liquid biopsies, and custom-designed drug delivery systems, in which exosomes are targeted toward malignant cells after being loaded with cytotoxic or immunomodulatory factors. Interference with cancer exosome release or blockage is a novel strategy that may prove to be an effective cancer treatment strategy. Due to their unique structural, and physicochemical properties such as low immunogenicity, efficient cellular entry, and capability to cross biological barriers as blood–brain barrier, exosomes are widely tested as drug carriers. Tumor-derived exosomes containing their oncogenic information are tested as tumor biomarkers in several malignancies as colorectal cancer, hepatocellular carcinoma, and brain tumors [20] and in imaging studies to detect tumors as they can be loaded with inorganic molecules [21], fluorescent molecules and others to enhance signal at the tumor site [22].

In therapy, exosomes act as effective delivery systems for si-RNAs and miRNAs because they can store RNA without degradation, maintain its stability in blood, cross blood–brain barrier and overcome off-target effects [20]. Exosomes can improve chemotherapy effectiveness through improving chemotherapy drug bioavailability, stability, limited crossing biological barriers’ ability and can overcome drug resistance [18]. For example, when exosomes expressing Interleukin 3 were loaded with either Imatinib or BCR-ABL siRNA, they inhibited chronic myeloid leukemia cells’ growth in vitro and in vivo [18, 23]. When exosomes were added to standard treatment of brain tumors, they improved their therapeutic efficacy through their high permeability, biosafety, and better targeting ability. Exosomes have a diverse role in immunotherapy as tumor-derived exosomes are used as tumor antigens to activate the immune system while immune cells derived exosomes can modulate TME and inhibit tumor immune escape [20], however, issues as efficient and economic production in adequate quantities prevent their clinical use [24].

## Role of miRNA

Malignant cells secrete proteins in TME that induce neurite outgrowth, initiating crosstalk between malignant cells and nerves. Moreover, nerves release neurotransmitters, neuropeptides, noncoding RNAs (as miRNA), neurotrophic factors and other proteins during the process of nerve injury and repair exerting their effect directly or after binding to their corresponding receptors expressed on malignant cells thus supporting both an inflammatory response and malignant cell movement toward peripheral nerves. MiRNAs are found in plasma, blood, urine, saliva, other body fluids and in tumor tissues. MiRNAs overexpression or downregulation is associated with various kinds of human carcinoma forming a bridge between malignant cells, stromal cells, and nerves in TME enabling malignant cells to modulate physiological processes and evade immune surveillance. MiRNAs overexpression or downregulation can either promote or inhibit cancer invasion and migration ability [25], for example miR-124 overexpression inhibits renal cell carcinoma invasion [26], while miR-141 downregulation enhances bladder cancer invasion and migration ability [27]. MiR-34a plays an important role in mesenchymal stem cells and CSC differentiation [28], is highly expressed in adult brain, is involved in various neurodevelopmental and neuropathological processes, regulates neural stem cell/progenitor cell differentiation and neurogenesis and its knockdown induces neural reprogramming, and promotes sensory neurogenesis [29].

Figure was reformatted according to submission guidelines and moved to the end of the manuscript with added figures.

## Impact of neuroimmune crosstalk on cancer therapy and research

Recently, cancer research has expanded exponentially beyond the study of malignant cells to include complex and extensive heterotypic interactions between cancer cells and TME. Cancer cells modulate stromal, immune, and endothelial cells enhancing proliferation, survival, and metabolic changes that support tumor growth and metastasis, recruit peripheral nerves to TME which drive tumor proliferation, invasion, metastasis, and immune evasion. The complex direct dynamic interplay between malignant cells, nerves, immune system, vasculature in TME or the indirect interactions through mediators have paved the way for novel strategies in cancer therapy and research. Developing therapies that can target the immune system or/and the nervous system as single agents or in combination with widely used therapies as chemotherapy, are novel therapeutic and research strategies, the following are examples of such strategies.

## Personalized immuno-oncology

Personalized immuno-oncology aims to control the immune system to eradicate cancer cells and prevent their spread. Immune checkpoint inhibitors (ICI) are monoclonal antibodies that target the PD-1 and PD-L1 interaction. PD-1 enables T cells to recognize cancer cells as foreign and prevent deactivation of an immune system response. PD-L1 is a predictive biomarker used to identify patients most likely to respond to ICI therapy whether used alone or combined with chemotherapy. Metastatic non-small cell lung cancer (NSCLC) patients who have PD-L1 ≥ 50% and negative for other actionable molecular markers (epidermal growth factor receptor mutation, anaplastic lymphoma kinase rearrangement and others) are offered first line ICI instead of chemotherapy after a randomized controlled trial demonstrated superior overall survival (OS) over chemotherapy [30].

## Antineurogenic therapy

Entrectinib (a selective tyrosine kinase inhibitor) was granted accelerated approval by the Food and Drug Administration (FDA) to be used as a single agent for pediatric patients 12 years or more, or adults with metastatic and/or inoperable solid tumors that have NTRK gene fusion without a known acquired resistance mutation, who progressed after treatment or have no evidence based standard therapy [31]. Based on phase 1–2 trials, Larotrectinib was approved for adult and children with TRK fusion—positive solid tumors due to its effectiveness [32].

Anti-NGF mAb therapy (tanezumab) ameliorates chronic pain especially neuropathic pain due to cancer and decrease adverse events experienced by conventional analgesics (e.g., opioids). Unfortunately, rapidly progressive osteoarthritis, joint fractures and neurological disorders were reported as side effects. The underlying molecular mechanisms for these side effects need to be elucidated. These side effects resulted in FDA halting of clinical trials using anti-NGF mAb therapy for a while then later resumed them with lower doses and not combined with nonsteroidal anti-inflammatory drugs [33, 34].

## Drug repurposing

To optimize cancer patient outcomes and minimize clinical and financial toxicities, repurposing of drugs that are already in use for other indications arises as an attractive strategy. βeta-blockers (used for treatment of cardiovascular diseases) are being used in cancer due to their antagonist action on the adrenergic system (through inhibition of b-AR) inhibiting tumorigenesis, angiogenesis, and tumor metastasis, suggesting a novel cancer therapeutic approach through drug repurposing [35].

In a meta-analysis of 36 studies involving 319,006 cancer patients, βeta-blocker use was not associated with improved OS, disease-free survival (DFS), progression-free survival (PFS), or recurrence-free survival (RFS), but significantly improved cancer-specific survival in patients with ovarian cancer, pancreatic cancer, and melanoma patients [36]. In another study, βeta-blocker use had no effect on complete response but was associated with improved DFS and OS in melanoma and ovarian cancer. On the other hand, its use was associated with a reduction in DFS and OS in endometrial cancer and a reduction in OS in head and neck, prostate cancer and lung cancer denoting that beta blockers are tumor specific [37]. Similarly, drugs that modulate the neural component within the TME as substance P (aprepitant, NK-1 antagonist), gabapentinoids, and eptinezumab, erenumab may be repurposed and used as adjuvant therapies [8].

To assess whether βeta-blockers improved outcomes when combined with ICI in cancer patients, a recent meta-analysis was conducted. The results showed that using βeta-blockers in combination with ICI was not associated with significant improvement in either OS or PFS [38]. In unresectable hepatocellular carcinoma patients treated with ICI combined with βeta-blockers had comparable OS and overall response rates between those who used βeta-blockers and those who did not [39].

A recent meta-analysis evaluated the use of βeta-blockers in combination with standard treatment for early-stage breast cancer patients. Subgroup analyses was done according to βeta-blocker use per breast cancer subtype (luminal, human epidermal growth factor receptor 2 (HER2)-positive or triple negative) and βeta-blocker class (nonselective or β1-selective). βeta-blocker use was associated with a significant RFS improvement in the whole population and in patients with triple-negative disease [35].

Although propranolol increased antitumor activity of trastuzumab by resensitizing tumor cells in preclinical trials [40], yet βeta-blockers use was associated with worse OS in patients receiving anti-HER2 therapy for advanced breast cancer regardless of any cardiovascular disease status as reported from a pool analysis of EMILIA, TH3RESA, MARIANNE, and CLEOPATRA clinical trials [41].

In lung cancer, βeta-blocker use was evaluated in a meta-analysis including ten retrospective cohort studies with 30,870 patients. Results showed that βeta-blockers use was not associated with significantly improved OS in lung cancer. Subgroup analysis showed similar results for NSCLC and small cell lung cancer, for use before and after lung cancer diagnosis, with or without adjustment of smoking. Improved OS was shown in stage III lung cancer and in patients who had not undergone surgery resection. Use of a non-selective βeta-blocker was associated with worse OS [42].

βeta-blockers reduced prostate cancer-specific mortality in a retrospective study involving 3561 prostate cancer patients with high-risk or metastatic disease [43] and was associated with prolonged survival in prostate cancer patients when added to conventional treatments in a meta-analysis [44].

Neurotransmitters provide a therapeutic potential to modulate immune cells’ function. Combination of neurotransmitter agonists or antagonists with other therapeutic modalities to suppress or stimulate immune responses is a novel strategy for cancer therapy. In a pilot study, propranolol was used as an adjuvant therapy for patients with epithelial ovarian cancer undergoing either chemotherapy or surgery. The results showed that it was associated with improved mood and significant reductions in proinflammatory IL-6 and IL-8 correlating with response to therapy [45]. In another study, propranolol demonstrated a significant inverse association with recurrence for stage IB to IIIA cutaneous melanoma patients when used as an off-label adjuvant treatment [46].

βeta-blockers (propranolol, propranolol hydrochloride) are being tested in pancreatic, breast, cervical, gastrointestinal cancer, colorectal, prostate cancer, melanoma, and locally recurrent/metastatic solid tumors: NCT02596867, NCT03919461, NCT03245554, NCT03838029, NCT02944201, NCT03152786, NCT01988831and NCT02013492. Neoadjuvant chemotherapy combined with propranolol are being tested in breast (NCT01847001) and gastric (NCT04005365) cancer patients [47]. Bethanechol, a selective muscarinic acetylcholine receptor agonist, is currently tested as adjuvant therapy for pancreatic cancer (NCT03572283) [47].

## MiRNA-based therapeutics

Research using miRNA therapeutics either through supplementing downregulated miRNA by synthetic oligonucleotides or silencing overexpressed miRNAs via artificial antagonists have been conducted recently. MiRNA therapeutics success will depend on solving pharmacokinetic issues (degradation of oligonucleotides by RNases), difficulty in identification of the best miRNA candidates for each disease type, and targeted delivery issues. Cell specific controlled delivery and safety profile of miRNA-based therapies has been improved after development of nanocarrier-based platforms [48].

## Si-RNA-based therapeutics

SiRNAs are small non-coding RNAs which can knock down expression of target genes by customizing gene sequence encoding a specific protein isotype thus silencing gene expression of different TME cells. A cocktail of siRNAs can downregulate various pathways simultaneously better than small molecule inhibitors or antibodies, which target “druggable” proteins. Therefore, siRNAs therapy is a promising strategy to jeopardize TME crosstalk. Although siRNAs have several advantages over small therapeutics drugs and monoclonal antibodies, i.e., they act through perfect base pairing with mRNA and any gene can be easily targeted by an siRNA, yet there are several barriers for their clinical application: poor pharmacokinetic properties, quick degradability by RNAses, induction of off-target effects and ability to trigger activation of Toll-like receptor 3 thus negatively influencing the immune system. To overcome siRNA stability and delivery problems, nanoparticles, siRNA-peptide conjugates, bioconjugation and aptamers are used as delivery systems [49].

A promising, novel, specific, and non-toxic approach is to use siRNA-mediated targeting of immune infiltrates. Although there are several FDA-approved monoclonal antibodies and cytotoxic drugs for inhibiting the pro-tumorigenic function of T regulatory cells [50], yet these approaches are not widely used in clinical practice. Although targeting the PD-1/PD-L1 pathway is currently widely used in clinical practice, yet resistance eventually develops paving the way to develop new personalized therapies in the context of PD-1/PD-L1+ tumors as siRNAs. Currently, there are no siRNA-based drugs approved for cancer therapy [51].

## Tumor denervation

Tumor denervation have been attempted surgically and chemically to block tumor growth and metastasis as a novel research avenue, however, there are still unresolved issues for using these methods clinically as degree, modes of innervation (which are different in different tumor tissues), factors involved in neurogenesis and axonogenesis at different stages of tumorigenesis, targeting tumor-specific pathways without affecting established neural circuits elsewhere in the body, nervous system reaction to manipulation chronology of tumor innervation, nature of tumor innervation at each stage of cancer development, a complete landscape of tumor-nerve content and function across various cancer types should be completely understood to identify the optimal time and mode of intervention. With a greater understanding of autonomic neuroanatomy and advances in imaging technologies, more precise denervation procedures whether surgically or pharmacologically can be developed. Also, tumor denervation can be used as an adjuvant treatment to improve the surgical success rate [52].

## Cellular therapies

**Fig. 2:**
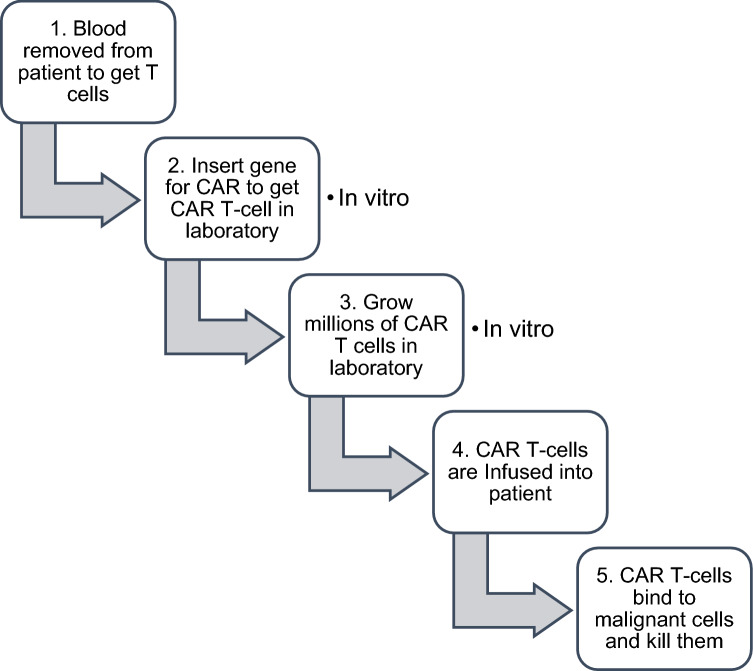
CAR T-cell therapy procedure (currently preparation of CAR T-cells is done in vitro, research is being done to prepare CAR T-cells inside patients)

In addition to Chimeric antigen receptor T-cell (CAR T-Cell) therapy (a human gene therapy using patients’ T cells used as single agents for treatment of hematogenous malignancies), Fig. 2, other T-cell sources are being tested such as T-cells induction by gene-editing technologies like CRISPR, nanotechnology and mRNA-based approaches to allow creation of CAR T-cells inside patients. Also, CAR NK cell therapies, TILs and T-cell receptor therapies are being tested. The TIL uses immune cells that recognize cancer cells with mutations specific to that cancer, while the T-cell use naturally occurring receptors that can recognize antigens inside tumor cells. Adoptive T cell therapy is being improved by using selected neurotransmitter agonists or antagonists for treating harvested TIL in vitro before transfer. Furthermore, neurotransmitter agonists or antagonists are injected into solid tumors to modulate NK and T cells. Such neuroimmunotherapy approaches provide novel research and therapeutic avenues. However, there are several challenges for using neurotransmitter receptors as they have multiple subtypes that can initiate different types of immune responses. So, they can enhance an immunostimulatory response in one immune subset and/or enhance an inhibitory response in another immune subset depending on the receptors’ type and involved signaling targets. Furthermore, the outcome of initiated immune response may differ according to concentration of neurotransmitters in circulation or in TME [53, 54].

## Oncolytic virus therapy

**Fig. 3:**
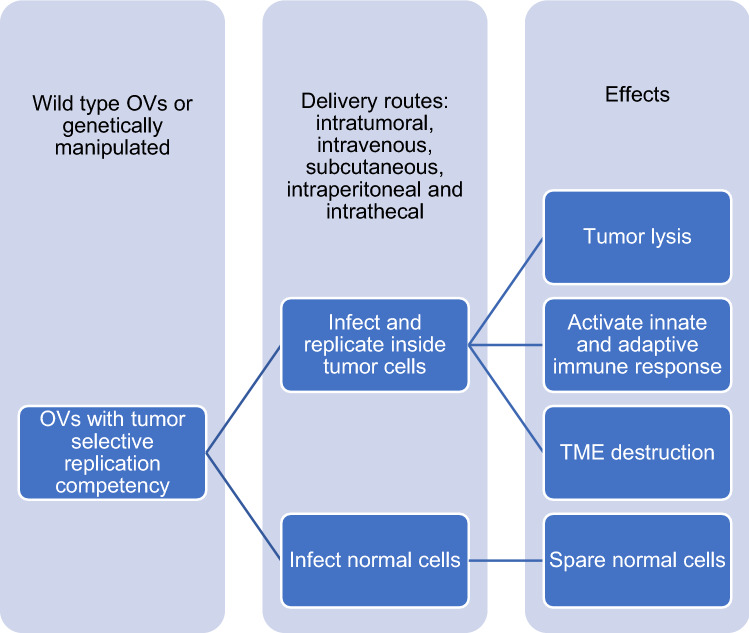
Oncolytic virus therapy as a novel therapy sparing normal cells

Oncolytic virus (OV) therapy or virotherapy is a novel strategy in cancer therapy. OVs replicate in malignant cells causing their death through a multimodal mechanism of action while sparing normal cells. Wild type or genetically modified viruses can activate innate and adaptive immune response, induce tumor cytotoxicity or trigger vasculature collapse as shown in Fig. 3 [55]. OVs are human and non-human, genetic modification is required for human viruses to improve specificity and safety, while it is required for both to improve efficacy [18].

OVs are used as single agents (an engineered oncolytic herpes simplex virus type 1 was approved for treatment of unresectable stages IIIB and IV melanoma) [56] or tested in combination with chemotherapy as cisplatin in mesothelioma cell lines [57] or combined with ICI **(**Onco-Immunotherapy) to increase response duration to these drugs, induce multiple immune pathways to halt cancer progression, upregulate PD-L1 to maximize ICI efficacy, sensitize patient’s immune repertoire for ICI and anti CTLA-4 therapies and provide a therapeutic option for patients who are unresponsive to conventional treatments. Synergy between OV therapy and ICI is supported by preclinical data, however there are challenges regarding timing of administration, different tumor types and different oncolytic virus types combined with ICI. Clinical trials testing oncolytic virus with ipilimumab (anti-CTLA-4) and pembrolizumab (anti-PD-1) are currently in progress [58]. Targeting CSC by OVs has emerged as a novel and effective strategy for glioma treatment to achieve long-term control. OVs are capable of destructing cancer cells as well as CSC in preclinical data [59]. Fast-track approval of several OVs (DNX-2401, Toca511, and PVS-RIPO) by the FDA were granted for brain tumors, after they showed durable complete responses in about 20% of patients and rare serious side effects [60]. Furthermore, four glioblastoma cases were reported to have received OVs after progression on standard therapy (as compassionate use) and achieved durable response, improved quality of life and long-term survival ranging between 4 and 14 years [61].

## Conclusion

As global cancer burden is expected to rise, novel diagnostic, prognostic and therapeutic modalities are needed. Neuroimmune crosstalk in cancer plays a critical role in tumorigenesis, invasion, proliferation, and metastases. The dynamic interplay between nerve, immune, and malignant cells within the TME remains to be fully understood. Based on the abovementioned display, various personalized therapies are used in clinical practice, for example ICI which are monoclonal antibodies that target PD-1 and PD-L1 interaction that may occur between cancer cells and immune cells or between cancer cells and nerve cells are widely used. Also, CAR T-cell therapy is used in treatment of adults with relapsed or refractory follicular lymphoma after two or more lines of systemic therapy, or in treatment of patients up to 25 years of age with B-cell precursor acute lymphoblastic leukemia that is refractory to treatment or in second or later relapses and for treatment of adult patients with relapsed or refractory large B-cell lymphoma [diffuse large B-cell lymphoma (DLBCL), high grade B-cell lymphoma and DLBCL arising from follicular lymphoma] after two or more lines of systemic therapy and lastly for multiple myeloma that progresses or is refractory after four prior treatments. In addition, selective TRK inhibitors such as Entrectinib and Larotrectinib are used as single agents for patients with solid tumors that harbor NTRK gene fusion. Finally, oncolytic virus therapy is used for treatment of unresectable stage IIIB or IV melanoma. Advances in genetic, molecular mapping, imaging, high-throughput single-cell analysis and computational systems biology will lead to a more comprehensive analysis of neuroimmune modulation. Targeting the neuroimmune communication is an exciting transdisciplinary area of cancer therapy and research.

## Data Availability

Datasets were not generated in this article. All data that support the findings in this study are included in the published article.

## Abbreviations

miRNA: Micro RNA
siRNAs: Small interfering RNAs
PNS: Peripheral nervous system
SNS: Sympathetic nervous system
TME: Tumor microenvironment
ECM: Extracellular matrix
MDSC: Myeloid-derived suppressor cells
PD-L1: Programmed death-ligand 1
PD-1: Programmed death-1
b-AR: β2-Adrenergic receptor
CSC: Cancer stem cells
NGF: Nerve growth factor
TILs: Tumor-infiltrating lymphocytes
NK: Natural killer
TRK: Receptor tyrosine kinase
NTRK: Neurotrophic receptor tyrosine kinase
VEGF: Vascular endothelial growth factor
NE: Norepinephrine
ICI: Immune checkpoint Inhibitors
OS: Overall survival
FDA: Food and Drug Administration
NSCLC: Non-small cell lung cancer
DFS: Disease-free survival
PFS: Progression-free survival
RFS: Recurrence-free survival
HER2: Human epidermal growth factor receptor 2
CAR T-Cell: Chimeric antigen receptor T-cell
OV: Oncolytic virus
CTLA-4: Cytotoxic T-lymphocyte-associated antigen 4
DLBCL: Diffuse large B-cell lymphoma
